# Effect of protic surfactant ionic liquids based on ethanolamines on solubility of acetaminophen at several temperatures: measurement and thermodynamic correlation

**DOI:** 10.1186/s13065-024-01243-x

**Published:** 2024-07-25

**Authors:** Parisa Akbarzadeh Gondoghdi, Hemayat Shekaari, Masumeh Mokhtarpour, Mirhesam Miraghazadeh Sardroud, Ramin Afkari, Mohammad Khorsandi

**Affiliations:** 1 Department of Physical Chemistry, University of Tabriz, Tabriz, Iran; 2 Research Center for Bioscience and Biotechnology, University of Tabriz, Tabriz, Iran

**Keywords:** Solubility, Acetaminophen, Protic ionic liquids, Ethanolamine, Modelling, Lauric acid

## Abstract

Absolute qualifications with the application of protic ionic liquids (PILs) and a recognition of the numerous thermophysical features of these materials are required in various processes. Due to the wonderful applications of these compounds and their high potential in the chemical and pharmaceutical industries, there is a particular eagerness to utilize these PILs in drug solubility and delivery area. The aim of this investigation was to explore the solubility of the acetaminophen (ACP) in three PILs base on ethanolamine laurate [(2-hydroxyethylammonium laurate [MEA]La), (bis(2-hydroxyethyl)ammonium laurate [DEA]La), and ( tris(2-hydroxyethyl)ammonium laurate [TEA]La)]. The shake flask method has been employed in this study, and the conditions were set at *T* = (298.15–313.15) K and atmospheric pressure. Moreover, the experimental solubility data was correlated using a variety of empirical and thermodynamic models, encompassing *e*-NRTL and Wilson activity coefficient models and the empirical models such as Van’t Hoff-Jouyban-Acree and Modified Apelblat-Jouyban-Acree. Their performance for the system containing [MEA]La follow the trend for activity coefficient models and empirical respectively: the Wilson > *e*-NRTL and Modified Apelblat–Jouyban–Acree > Van’t Hoff–Jouyban–Acree. On the other hand, [DEA]La and [TEA]La PILs followed slightly different trend for activity coefficient models and empirical respectively: the Wilson > *e*-NRTL and Van’t Hoff–uyban–Acree > Modified Apelblat–Jouyban–Acree. The Van’t Hoff and Gibbs equations were used to determine the thermodynamic properties of dissolution in the studied systems.

**Supplementary Information:**

The online version contains supplementary material available at 10.1186/s13065-024-01243-x.

## Introduction

Drug formulation is a crucial factor for an appropriate drug dosage. Numerous studies have been performed to enhance the solubility of various active pharmaceutical components in order to produce homogenous pharmaceutical dosage forms, as many pharmaceuticals have low water solubility. Thus, a wide range of techniques have been implemented, involving pH modification, co-solvency, cyclodextrins, solid dispersions, and complexation [1]. Among these methods, co-solvency is widely used due to its convenience and low cost, wherein the solvent power of the primary solvent is increased by adding a tiny amount of a secondary solvent [2].

**Fig. 1:**
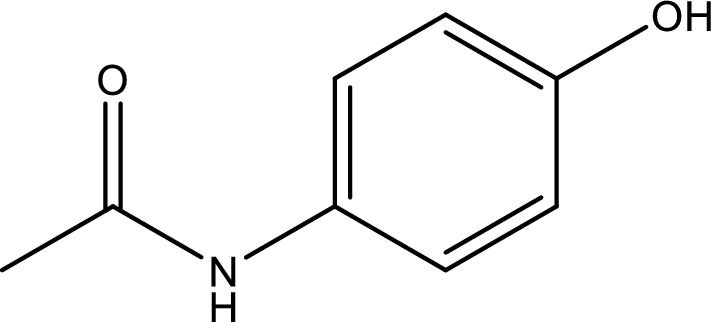
The 2D- molecular ctructure of the ACP

The choice of solvents is crucial to control possible chemical reactions and for the purification process and the final drug formulation. Despite the fact that the pharmaceutical industry frequently employs classic organic solvents including methanol, alcohols, chloroform, and ethyl acetate, overuse of these volatile and dangerous solvents can affect the environment, and there is rising pressure to minimize their usage. To address this issue, scientists are working to replace these organic solvents with non-volatile more environmentally friendly systems, such as proticionic liquids (PILs) and low-melting mixtures (LMMs) [3]. Ionic liquids are appealing as sustainable solvents since they present lower flammability and vapor pressure than volatile organic compounds (VOCs) and can be formulated to be biodegradable and to present lower toxicity [4, 5]. In particular, protic ionic liquids (PILs) which are generated by the Brønsted acid–base reaction (Fig. 1), can participate in appropriate chemical interactions, encompassing proton donation and acceptance, which might affect the solubility of drugs. Furthermore, PILs have a lot of potential for industrial applications due to their simple manufacturing and frequently involve inexpensive or even renewable reactants. The properties of PILs, including solubility, can be tailored by a careful choice of the proton acceptor and proton donor and is an important task when designing their properties for drug formulations. Additionally, Considering they are inexpensive and environmentally safe, ethanol-amine based protic ionic liquids have attracted a lot of research. Numerous studies have been conducted on these materials with the goal of enhancing medicinal products. As a result, new medication formulations and delivery methods using these PILs have been found. These substances are noteworthy for having non-toxic qualities, which makes them appropriate for use as cryoprotectant agents for mammalian cell lines [6, 7].

Lauric acid, is an edible saturated fatty acid that is present in a variety of plants life, encompassing coconut oil and other dietary supplements [8–10]. On the other hand, alkanolamines, including mono-, di-, and tri-ethanolamine, can be discovered in phospholipid biological membranes naturally and are frequently employed as “scrubbers” in factories to remove acidic impurities as hydrogen sulfide or carbon dioxide. They may additionally act as the foundation material for PIL production. Due to their capacity to function as a thickening, emulsifying, and detergent agents in various formulations, their combination with fatty acids yields surfactants, which are employed in the formulation of health care products and cosmetics [11]. PILS have also shown their ability to improve drug absorption by altering their processing and physical characteristics, particularly, solubility and controlled release of the drug.Acetaminophen (ACP), is a widely used drug that plays an important role in the pharmaceutical industry as it is a prominent painkiller and antipyretic in current treatments [12].

The current investigation builds on our previous work [13–17], which explored the use of green solvents, including PILs as co-solvents. In this work, we focused on understanding how protic ILs based on alkanolamines and lauric acid affects the solubility of ACP. In particular, three different PILs, namely ((2-hydroxyethylammonium laurate [MEA]La), (bis(2-hydroxyethyl)ammonium laurate [DEA]La), and [tris(2-hydroxyethyl)ammonium laurate [TEA]La)] have been used to evaluate their impact on ACP solubility at different temperatures and various PIL concentrations. Furthermore, the solubility data using several thermodynamic models, the activity coefficient and emprical soulubility models including the Van't Hoff-Jouyban-Acree model and Modified Apelblat-Jouyban-Acree model [18, 19]. Ultimately, the apparent thermodynamic properties of ACP dissolution in the systems under investigation have been determined by utilizing the Van't Hoff and Gibbs equations. This study contributes to the ongoing research on green solvents and provides valuable insights for the pharmaceutical industry to explore alternative solvents that are environmentally friendly and sustainable.

## Material and methods

### Materials

The chemicals were purchased from the following companies: ethanol from Sigma-Aldrich, lauric acid from Merck Co. the monoethanolamine (2-hydroxyethylamine, MEA), diethanolamine (bis(2-hydroxyethylamine), DEA), triethanolamine (tris(2-hydroxyethylamine),TEA) from Shazand pterochemical Co. and acetaminophen (ACP) from Dana. The each one of the chemicals which had a purity weight fraction greater than 0.99 in mass percent. The solutions were prepared using freshly doubly distilled deionized water. The relevant information about the chemicals used in the study, including chemical name, their sources, CAS numbers, mass fraction (purities) are provided in Table 1.

**Table 1:** The comprehensive description of the chemicals name, provenance, CAS number, purity, and structure

Chemical name	Provenance	CAS No	Mass fraction (^a^purity)	Structure
Acetaminophen (ACP)	Merck	103-90-2	> 0.99	
Monoethanolamine (2-hydroxyethylamine, MEA)	Shazand petrochem	141-43-5	> 0.99	c
Diethanolamine (Bis-2-hydroxyethylamine, DEA)	Shazand petrochem	111-42-2	> 0.98.5	
Triethanolamine (Tris-2-hydroxyethylammonium, TEA)	Shazand petrochem	102-71-6	> 0.99	
Lauric acid	Merck	143-07-7	> 0.98	

^a^ The purities were provided by the suppliers

### Protic ionic liquid synthesis

The present study involved the synthesis and purification of protic ionic liquids (PILs) using a neutralization method. Encompassing MEA, DEA and TEA with lauric acid were used as starting materials to synthesize PILs. The synthesis process involved stirring the ethanolamines in a three-neck glass flask, then gradually adding lauric acid using a dropping funnel while stirring at room temperature. In order to dry the synthesized PILs, a vacuum pump was employed for a duration of 3 h with up to 1 kPa. This process was undertaken to ensure purity of the resulting product and maintain its quality and effectiveness. The shortened version of these processes has been presented in Table 2. These procedures ultimately lead us to achieve a high yield of about > 98%. The aforementioned method offers a simple way to create PILs with specifc features for a variety of applications [20]. To determine the water contents of the synthesized PILs, the Karl − Fisher titration technique (method TitroLine KF) was applied.

**Table 2:** Physicochemical properties of the protic ionic liquids employed in this study at 298.15 K and 866 hPa^a^

PILs	*M*_PIL_ (g∙mol^−1^)	Purification method	Analysis method	Water content (ppm)
2-hydroxyethylammonium laurate [2-HEA]Lau]	261.40	Extraction/filtration/rotary evaporator	FT-IR-H NMR	179
bis(2-hydroxyethyl)ammonium laurate [2-HDEA]Lau]	305.46	Extraction/filtration/rotary evaporator	FT-IR-H NMR	185
tris(2-hydroxyethyl)ammonium laurate [2-HTEA]Lau]	349.51	Extraction/filtration/rotary evaporator	FT-IR-H NMR	198

^*a*^ Standard uncertainty for u*(T)* = 0.1 K and u*(P)* = 10 hPa

### Solubility determination

**Fig. 2:**
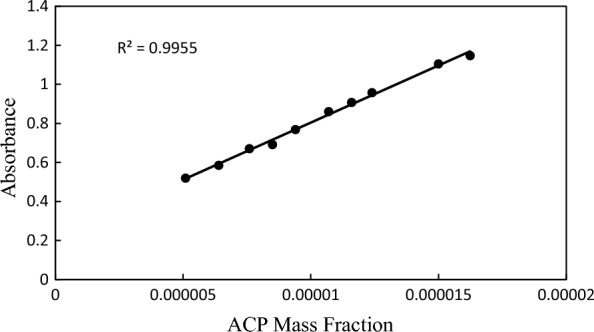
The calibration curve for acetaminophen (ACP)

A calibration curve for ACP was initially generated before the solubility amounts were determined (Fig. 2). The calibration curve for the experiment was obtained by dissolving precise amounts of ACP in a solution of ethanol and distilled deionized water using a double-beam UV–vis spectrometer (T80 Japan) [21]. The experimental solubility results were determined using a variety of techniques, including the shake-flask method [22]. Aqueous binary mixtures based on PILs have been made utilizing an analytical balance with a 10^–4^ g precision for determining the solubility results. Glass vials with a specific amount of water + PILs were filled with the extra acetaminophen. Following that, the samples were combined for 3 h and left for three days in a water bath thermostat (ED, Julabo Co., Germany) with temperature control and an uncertainty of 0.01 K. To separate the liquid and solid phases, a Hettich D-7200 centrifuge was employed (with 4000 RPM at room temperature at 10 min). An ethanol + water solution was applied to dilute the liquid phase after the saturated solutions were filtered through a 0.22 m PTFE filter. Subsequently, the drug concentration in the solutions was ascertained using a calibration curve and a UV–vis spectrometer at 243 nm [23].

The amount of drug that dissolves in a mixture of water and the drug, a mixture of water and ACP, or a mixture of water and protic ionic liquids (PILs) was calculated using Eq. 1 [24, 25]:

1 $$x_{1} = \frac{{\frac{{w_{1} }}{{M_{1} }}}}{{\frac{{w_{1} }}{{M_{1} }} + \frac{{w_{2} }}{{M_{2} }} + \frac{{w_{3} }}{{M_{3} }}}}$$

where *M*_i_ and *W*_i_, respectively, indicate the weight fraction and molar mass of each component (i) in systems.

### Solubility models

Thermodynamic models are important for determining the solubility of drugs in different solvents. This is a critical parameter in the pharmaceutical industry, as it affects the delivery and absorption of drugs. Thermodynamic models use equations of equilibrium to calculate the solubility of a drug in different solvents. One important factor in these models is the excess molar Gibbs energy (G^ex^) [26]. The G^ex^ is a measure of the non-ideal behavior of a mixture of solvents. Another important factor in thermodynamic models is local composition theory. The short-range order and nonrandom molecule orientations caused by changes in molecular size are explained by this theory. The activity of the solute in the saturated solution has to exceed the activity of the solute in its pure solid-state form in order to determine the solubility of a solute in a solution at a specific temperature. Equation 2 states that this could be accomplished by employing a solid–liquid equilibrium (SLE) framework to control the solute's activity in the saturated solution [45]:

2 $$\ln x_{1} = - \ln \gamma_{1} + \frac{{\Delta_{fus} H}}{R}(\frac{1}{{T_{{m_{1} }} }} - \frac{1}{T}) - \frac{1}{RT}\int_{{T_{{m_{1} }} }}^{T} {\Delta C_{P1} dT} + \frac{1}{R}\int_{{T_{{m_{1} }} }}^{T} {\frac{{\Delta C_{P1} }}{T}dT}$$

where *T* and ${{T_{{m_{1} }} }}$ are the experimental and melting temperatures, respectively, and R is the gas constant; $\Delta_{fus} H$, $\gamma_{1}$ and $\Delta C_{P1}$ relates to the enthalpy of fusion (26.0 KJol. Mol ^−1^[27]), activity coefficient, and the variation in molar heat capacity between ACP's melting and solid states. Subsequently suitable simplification [28], the simplified equation became as follows:

3 $$\begin{gathered} \ln x_{1} = \frac{{\Delta_{fus} H}}{R}(\frac{1}{T}_{m} - \frac{1}{T}) - \ln \gamma_{1} \hfill \\ \hfill \\ \end{gathered}$$

The melting temperature (*T*_*m*_), activity coefficient (γ), and enthalpy of fusion (ΔH_fus_) of ACP must be known in order to assess its solubility in various solvents. This project correlated the experimental solubility data for ACP (x_1_), which means that it found a mathematical relationship between the solubility of ACP and the three factors listed above. To discribe the solubility data, activity coefficient-based models used to describe the solubility data by combining the evaluated information from Eq. 3.

The molar excess Gibbs energy (G^ex^) is shown to be the sum of two contributions in order to try to extend the *e*-NRTL and Wilson models for systems with many components that include electrolytes in the aqueous solution:

4 $$\frac{{G^{ex*} }}{RT} = \frac{{G^{ex*,LR} }}{RT} + \frac{{G^{ex*,SR} }}{RT}$$

where the asymmetric convention is indicated by the superscript ex*, short- and long-range interactions are indicated by the letters SR and LR, respectively. Pitzer [29], developed the expanded Pitzer-Debye Hückel (Gex) PDH model, which may be employed to model interactions with long-range terms. Short-range interactions have also been modeled utilizing other models, including Wilson and *e*-NRTL.

### The *e*-NRTL model

A novel method for determining the activity coefficient of each species in a solution was developed by Chen (1982), Chen and Evans (1986), and it was based on the electrolyte Nonrandom Two-Liquid (*e*-NRTL) model [30, 31].

The Pitzer–Debye–Hückel contribution, which takes into consideration long-range interactions between ions, and the Nonrandom Two-Liquid (*e*-NRTL) model contribution, which takes into account short-range interactions between all species, add up to the activity coefficient. Using the Nonrandom Two-Liquid (*e*-NRTL) model, one can utilize Eqs. 5 and 6 to determine the activity coefficient of species γ_1_ in a solution:

5 $$\ln (\gamma_{i}^{*} ) = \begin{array}{*{20}l} {\ln (\gamma_{i}^{*PDH} )} \hfill \\ \end{array} + \ln (\gamma_{i}^{*NRTL} )$$

6 $$\ln \gamma_{i} = \frac{{\sum\limits_{j = 1}^{m} {\tau_{ij} G_{ij} x_{j} } }}{{\sum\limits_{i = 1}^{m} {G_{li} x_{l} } }} + \sum\limits_{j = 1}^{m} {\frac{{x_{j} G_{ij} }}{{\sum\limits_{l = 1}^{m} {G_{lj} x_{l} } }}\left( {\tau_{ij} - \frac{{\sum\limits_{r = 1}^{m} {x_{r} \tau_{rj} G_{rj} } }}{{\sum\limits_{l = 1}^{m} {G_{lj} x_{l} } }}} \right)}$$

where $G_{ij}$ were definition as $G_{ij} = \exp ( - \alpha_{ij} \tau_{ij} )$, $\tau_{ii} = \tau_{jj} = 0$ and $\alpha_{ij} = \alpha_{ji}$.

Furthermore, calculating the binary interaction parameter ($\tau_{ij}$) has been done by Eq. 7:

7 $$\tau_{ij} = \frac{{g_{ij} - g_{ii} }}{RT}$$

the *g*_*ij*_ is an energy parameter characteristic of the *i–j* interactions.

### The Wilson model

The Wilson model is a thermodynamic model which can be implemented for calculating activity coefficients in solutions. This framework considers both temperature and solution composition during evaluating activity coefficients:

8 $$\ln \gamma_{i} = 1 - \ln (\sum\limits_{j = 1}^{n} {(\Lambda_{ij} x_{j} ) - \sum\limits_{k = 1}^{n} {(\frac{{\Lambda_{ki} x_{k} }}{{\sum\limits_{j = 1}^{n} {(\Lambda_{kj} x_{j} )} }}))} }$$

where the binary interaction parameter, $\Lambda_{ij}$, is a measure of how the interactions between two different molecules in a mixture differ from the ideal interactions. It is based on the characteristic energy, $\lambda$, and molar volumes, $\upsilon$, of the solute and solvents. Equation 9 can be used to calculate the binary interaction parameter:

9 $$\Lambda_{ij} = \frac{{\upsilon_{j} }}{{\upsilon_{i} }}\exp \left( { - \frac{{\lambda_{ij} - \lambda_{ii} }}{RT}} \right)$$

### Van’t Hoff—Jouyban—Acree model

A different framework that can be utilized to clarify the relationship between the absolute temperature and the natural logarithm of a solute's mole fraction solubility in a solution is the Van’t Hoff equation:

Here is a more detailed explanation of the key terms:

10 $$\ln x_{T} = A + \frac{B}{T}$$

11 $$\log X_{1,T} = w_{2} (A_{2} + \frac{{B_{2} }}{T}) + w_{3} (A_{3} + \frac{{B_{3} }}{T}) + \frac{{w_{2} w_{3} }}{T}\sum\limits_{i = 0}^{2} {J_{i} (w_{2} - w_{3} )^{i} }$$

*he model parameters are A*_*2*_,*B*_*2*_,*A*_*3*_*, B*_*3*_,*and J*_*i*_.

### Modified Apelblat–Jouyban–Acree model

The relationship between temperature and solubility might be investigated with the semi-empirical Modified Apelblat model [32, 33]:

12 $$\ln x_{T} = A + \frac{B}{T} + C\ln T$$

The solubility of ACP in particular mixed solvents at various temperatures might be assessed with the Modified Apelblat-Jouyban-Acree model, a thermodynamic model. It is based on the mole fraction solubility of ACP in the selected mixed solvents at temperature T, which is indicated by x_T_, and the three equation parameters, A, B, and C [34]

13 $$\log X_{1,T} = w_{2} (A_{2} + \frac{{B_{2} }}{T} + C_{2} \ln T) + w_{3} (A_{3} + \frac{{B_{3} }}{T} + C_{3} \ln T) + \frac{{w_{2} w_{3} }}{T}\sum\limits_{i = 0}^{2} {J_{i} (w_{2} - w_{3} )^{i} }$$

The activity coefficients of the experimental and calculated states are denoted by $\ln \gamma_{i}^{\exp }$ and $\ln \gamma_{i}^{cal}$, respectively, and the number of experimental points could be represented by N. The relative deviation percent (ARD%) for the models already outlined may have been calculated utilizing Eq. 14 to determine the discrepancy between the calculated and experimental solubility data:

14 $${ARD}{\%} = 100(\frac{{\sum\limits_{i = 1}^{N} {\frac{{\left| {x_{i}^{\exp } - x_{i}^{cal} } \right|}}{{\left| {x_{i}^{\exp } } \right|}}} }}{N})$$

### Thermodynamic dissolution properties

The Gibbs and van der Hoff equations were used to determine the thermodynamic parameters of dissolution. A mean harmonic temperature of 305.55 K was utilized for the calculations, which were based on a temperature range of 298.15 K to 313.15 K [35]. The ACP standard molar enthalpy of dissolution,$\Delta H_{{{\text{soln}}}}^{o}$ was obtained by Eq. 15 [36–38]:

15 $$\Delta H_{so\ln }^{o} = - R(\frac{{\partial \ln x_{1} }}{{\partial ({\raise0.7ex\hbox{$1$} \!\mathord{\left/ {\vphantom {1 T}}\right.\kern-0pt} \!\lower0.7ex\hbox{$T$}})}})_{P}$$

where *T* is the absolute temperature and R (8.314 J∙K-1∙mol-1) is the universal gas constant [39] The mole fraction of ACP was indicated by*x*_1_. On the other hand, $$\Delta H_{so\ln }^{o}$$ can be calculated by plotting *lnx*_*1*_ versus $${\raise0.7ex\hbox{$1$} \!\mathord{\left/ {\vphantom {1 T}}\right.\kern-0pt} \!\lower0.7ex\hbox{$T$}} - {\raise0.7ex\hbox{$1$} \!\mathord{\left/ {\vphantom {1 {T_{hm} }}}\right.\kern-0pt} \!\lower0.7ex\hbox{${T_{hm} }$}}$$ which is called van’t Hoff plot:

16 $$\Delta H_{so\ln }^{o} = - R(\frac{{\partial \ln x_{1} }}{{\partial ({\raise0.7ex\hbox{$1$} \!\mathord{\left/ {\vphantom {1 T}}\right.\kern-0pt} \!\lower0.7ex\hbox{$T$}} - {\raise0.7ex\hbox{$1$} \!\mathord{\left/ {\vphantom {1 {T_{hm} }}}\right.\kern-0pt} \!\lower0.7ex\hbox{${T_{hm} }$}})}})_{P}$$

17 $$\Delta G_{so\ln }^{o} = - RT_{hm} \times {\text{ intercept}}$$

By solving Eqs. 16 and 17, we can determine the values of the parameters $\Delta H_{so\ln }^{o}$ and $\Delta G_{so\ln }^{o}$. The standard molar entropy of dissolution, $\Delta S_{so\ln }^{o}$, can then be calculated using the following equation [40–42]:

18 $$\Delta S_{so\ln }^{o} = \frac{{\Delta H_{so\ln }^{o} - \Delta G_{so\ln }^{o} }}{{T_{hm} }}$$

The relative contributions of entropy and enthalpy to the standard molar Gibbs free energy of dissolution of ACP are explained by Eqs. 17, 18, as shown by the symbols $\xi_{H}$ and $\xi_{{_{TS} }}$ , respectively [43]:

19 $${{\% }}\xi_{H} = \frac{{\left| {\Delta H_{so\ln }^{o} } \right|}}{{\left| {\Delta H_{so\ln }^{o} } \right| + \left| {T\Delta S_{so\ln }^{o} } \right|}} \times 100$$

20 $${{\% }}\xi_{TS} = \frac{{\left| {T\Delta S_{so\ln }^{o} } \right|}}{{\left| {\Delta H_{so\ln }^{o} } \right| + \left| {T\Delta S_{so\ln }^{o} } \right|}} \times 100$$

### Characterization of the prepared PILs

#### FT-IR spectrum of the synthesized PILs

Figures S1 − S3 presents the FT-IR spectra of three synthesized PILs (protic ionic liquids): 2 hydroxyethylammonium laurate [MEA]La, bis- 2 hydroxyethylammonium laurate [DEA]La, and tris- 2 hydroxyethylammonium laurate [TEA]La. Each PIL has its specifc set of FT-IR index peaks, which are listed as follows. For 2 hydroxyethylammonium laurate [MEA]La, the FT-IR peaks (KBr, cm^−1^) are observed at 530.81, 593.21, 720.25, 869.95, 924.69, 1013.10, 1047.01, 1077.01, 1135.19, 1315.58, 1413.11, 1465.85, 1548.57, 1644.86, 1720.87, 2851.75, 2921.02, and 3295.55. For bis-2 hydroxyethylammonium laurate [DEA]La, the FT-IR peaks (KBr, cm^−1^) appeared at 566.69, 720.48, 806.46, 965.67, 1013.10, 1047.01, 1064.36, 1407.65, 1463.61, 1560.29, 1620.98, 1724.41, 2851.72, 2921.58, and 3353.54. For tris-2-hydroxyethylammonium laurate [TEA]La, the FT-IR peaks (KBr, cm^−1^) are observed at 531.04, 566.69, 718.37, 915.24, 1031.92, 1082.54, 1408.08, 1468.05, 1562.47, 2852.12, 2920.76, 3151.51, and 3357.97. Comparing Figures S1 − S3 reveals some signifcant features. The peaks at 566 and 719 cm^−1^ indicate the presence of the COO − functional group. Additionally, the strong characteristic peaks at 2849 and 2920 cm^−1^ correspond to the antisymmetric and symmetric stretching vibrations of the CH2 groups, respectively, suggesting the presence of a long chain alkyl group. The presence of carboxylic acid carbonyls is evident from the peak at 1709 cm^−1^ (C = O peak). The antisymmetric stretching vibration peaks of CH3 are observed at 1094 cm^−1^, appearing as two weak peaks. Peaks around 914, 1468, and 1253 cm^−1^ represent the in-plane bending, symmetric, and out-ofplane bending vibrations, respectively, of the fatty acid carboxyl (COOH) groups [44]. The presence of a peak around 3357 cm^−1^ in the spectra corresponds to the N − H bond, which is a strong bond, and can be observed in PILs [45].

#### H NMR spectrum of the synthesized PILs

Figures S4 − S6 present the H NMR spectra of three synthesized PILs: 2 hydroxyethylammonium laurate [MEA]La, bis-2 hydroxyethylammonium laurate [DEA]La, and tris-2 hydroxyethylammonium laurate [TEA]La. Each PIL has its specifc set of H NMR index peaks, which are listed as follows. For 2 hydroxyethylammonium laurate [MEA]La, the H NMR peaks are observed at (500 MHz, DMSO) δ 6.15 (s, 3H), 3.51 − 3.45 (m, 2H), 2.74 − 2.66 (m, 2H), 1.99 (q, J = 7.4 Hz, 2H), 1.46 − 1.39 (m, 2H), 1.23 (s, 16H), 0.85 (t, J = 6.5 Hz, 3H). For bis-2-hydroxyethylammonium laurate [DEA]La—[Lau], the H NMR peaks are observed at (500 MHz, DMSO) δ 5.53 (s, 1H), 3.49 (ddt, J = 8.6, 5.5, 3.0 Hz, 6H), 2.68 (dt, J = 5.7, 2.9 Hz, 4H), 2.08 (ddd, J = 7.5, 4.7, 2.9 Hz, 2H), 1.45 (t, J = 7.2 Hz, 2H), 1.23 (s, 16H), 0.86 − 0.84 (m, 3H). For tris-2 hydroxyethylammonium laurate [TEA]La, the H NMR peaks are observed at (500 MHz, DMSO) δ 5.40 (s,1H), 5.02 (s, 3H), 4.32 (s, 6H), 2.50 (s, 6H), 2.01 (s, 2H), 1.43 (s, 2H), 1.23 (s, 16H), 0.85 (s, 3H). By analyzing the H NMR spectra, it has been noted that the synthesized PILs in this study exhibit a purity of surpassing 98% approximately [46].

### Results of the solubility

**Fig. 3:**
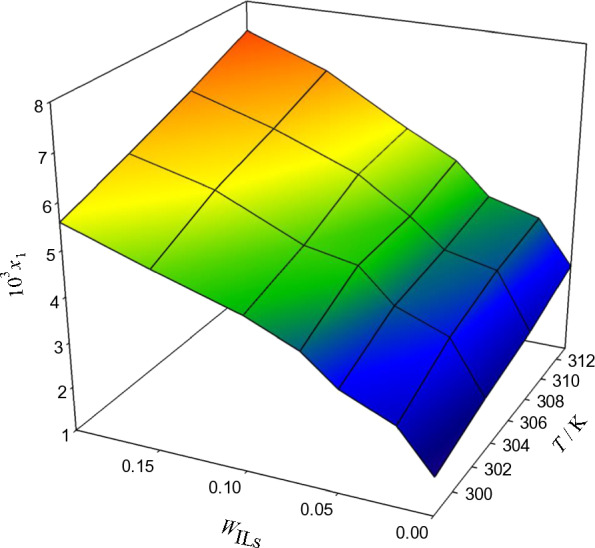
The relationship between temperature, weight fraction of PILs, w_P__ILs_, and mole fraction x_1_ in aqueous [MEA]La solutions and the solubility of ACP

**Fig. 4:**
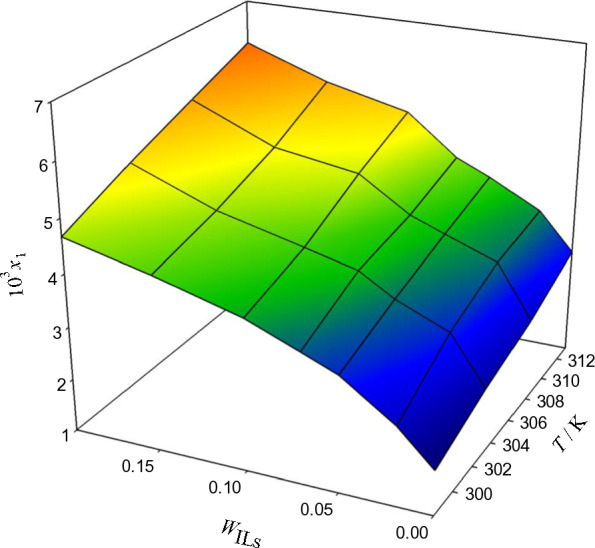
The relationship between temperature, weight fraction of PILs, w_P__ILs_, and mole fraction x_1_ in aqueous [DEA]La solutions and the solubility of ACP

**Fig. 5:**
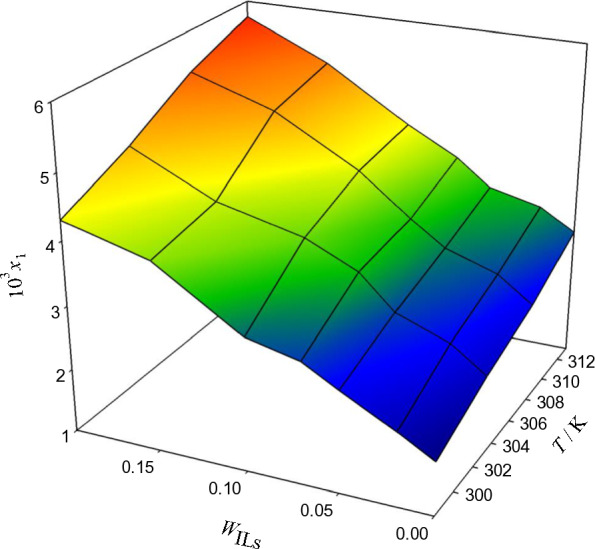
The relationship between temperature, weight fraction of PILs, w_P__ILs_, and mole fraction x_1_ in aqueous [TEA]La solutions and the solubility of ACP

Three protic ionic liquids, [MEA] La, [DEA] La, and [TEA] La, were present in binary solutions intended for examining the solubility of acetaminophen (ACP). Table 3 and Figs. 3, 4, and 5 illustrate the various temperatures (298.15 K, 303.15 K, 308.15 K, and 313.15 K) at which the experiments were carried out. Based on the evaluated experimental solubility, the results show that as the temperature and weight fraction of systems containing the protic ionic liquids increased, the dissolution of ACP is larger. The results indicate that the solubility of ACP increases with temperature and weight fraction of systems containing protic ionic liquids, based on the evaluated experimental data. According to the size of the cation in the surfactant molecule structure, one could suppose that the bilayers that result from monoethanolammonium laurate are more packed with stronger intermolecular hydrogen bonding networks than the diethanolammonium laurate and triethanolammonium laurate structures, whose larger cation decreases the self-assembly ability. In particular, the solubility of ACP in the [MEA]La PIL at each temperature is higher than in [DEA]La and [TEA]La PILs [47].

**Table 3:** The experimental ($x_{1}^{\exp }$)^a^ and calculated ($x_{1}^{cal}$) solubilities of acetaminophen (ACE) in the aqueous protic ionic liquid (PIL) solutions at different temperatures (T), PIL mass fraction in the aqueous mixture (w3), and pressure (dp = 866 hPa), as predicted by the Wilson and *e*-NRTL models, are presented

*T/*K		Wilson model		*e*− NRTL model	
	10^5^*x*_*1*_^exp^	10^5^*x*_1_^cal^	$100\frac{{x_{1}^{\exp } - x_{1}^{cal} }}{{x_{1}^{\exp } }}$	10^5^*x*_1_^cal^	$100\frac{{x_{1}^{\exp } - x_{1}^{cal} }}{{x_{1}^{\exp } }}$
ACP (1) + water (2) + ([MEA]La) (3)					
*w*_3_ = 0.0000					
298.15	1.839	1.840	− 0.04	1.847	− 0.46
303.15	2.239	2.243	− 0.16	2.253	− 0.61
308.15	2.545	2.548	− 0.10	2.556	− 0.44
313.15	3.009	3.013	− 0.13	3.021	− 0.41
*w*_3_ = 0.0200					
298.15	2.750	2.744	0.20	2.712	1.35
303.15	3.417	3.410	0.19	3.397	0.57
308.15	3.802	3.790	0.33	3.774	0.75
313.15	4.007	4.001	0.17	3.999	0.22
*w*_3_ = 0.0500					
298.15	3.256	3.261	− 0.18	3.233	0.70
303.15	3.861	3.868	− 0.17	3.835	0.68
308.15	4.055	4.060	− 0.13	4.024	0.75
313.15	4.320	4.327	− 0.16	4.293	0.63
*w*_3_ = 0.0700					
298.15	3.884	3.881	0.07	3.888	− 0.11
303.15	4.588	4.585	0.07	4.642	− 1.17
308.15	4.675	4.676	− 0.03	4.677	− 0.06
313.15	5.025	5.025	0.01	5.037	− 0.23
*w*_3_ = 0.1000					
298.15	4.380	4.381	− 0.03	4.401	− 0.49
303.15	4.807	4.817	− 0.20	4.827	− 0.41
308.15	5.407	5.403	0.06	5.454	− 0.88
313.15	5.600	5.603	− 0.06	5.625	− 0.44
*w*_3_ = 0.1500					
298.15	4.962	4.969	− 0.14	4.975	− 0.27
303.15	5.658	5.659	− 0.02	5.712	− 0.97
308.15	6.128	6.129	− 0.01	6.158	− 0.48
313.15	6.665	6.664	0.02	6.716	− 0.76
*w*_3_ = 0.2000					
298.15	5.589	5.587	0.04	5.557	0.58
303.15	6.123	6.123	0.01	6.096	0.43
308.15	6.695	6.697	− 0.03	6.641	0.80
313.15	7.337	7.339	− 0.03	7.308	0.40
ACP (1) + water (2) + ([DEA]La) (3)					
*w*_3_ = 0.0000					
298.15	1.839	1.839	− 0.46	1.849	− 0.52
303.15	2.239	2.240	− 0.61	2.241	− 0.08
308.15	2.545	2.545	− 0.44	2.547	− 0.09
313.15	3.009	3.010	− 0.41	3.014	− 0.16
*w*_3_ = 0.0200					
298.15	2.492	2.490	1.35	2.462	1.19
303.15	3.159	3.152	0.57	3.135	0.76
308.15	3.555	3.549	0.75	3.534	0.58
313.15	3.722	3.718	0.22	3.693	0.77
*w*_3_ = 0.0500					
298.15	3.181	3.179	0.70	3.161	0.64
303.15	3.576	3.576	0.68	3.581	− 0.13
308.15	3.917	3.919	0.75	3.903	0.36
313.15	4.227	4.227	0.63	4.217	0.24
*w*_3_ = 0.0700					
298.15	3.452	3.457	− 0.11	3.443	0.27
303.15	3.977	3.974	− 1.17	3.976	0.03
308.15	4.167	4.171	− 0.06	4.159	0.19
313.15	4.514	4.516	− 0.23	4.517	− 0.09
*w*_3_ = 0.1000					
298.15	3.849	3.852	− 0.49	3.856	− 0.17
303.15	4.220	4.222	− 0.41	4.236	− 0.39
308.15	4.793	4.788	− 0.88	4.829	− 0.75
313.15	5.266	5.258	− 0.44	5.271	− 0.10
*w*_3_ = 0.1500					
298.15	4.290	4.292	− 0.27	4.301	− 0.27
303.15	4.611	4.615	− 0.97	4.638	− 0.59
308.15	5.038	5.043	− 0.48	5.030	0.15
313.15	5.633	5.634	− 0.76	5.642	− 0.17
*w*_3_ = 0.2000					
298.15	4.669	4.664	0.58	4.666	0.07
303.15	5.223	5.214	0.43	5.175	0.92
308.15	5.710	5.708	0.80	5.689	0.37
313.15	6.188	6.185	0.40	6.158	0.48
ACP (1) + water (2) + ([TEA]La) (3)					
*w*_3_ = 0.0000					
298.15	1.839	1.844	− 0.29	1.860	− 1.15
303.15	2.239	2.247	− 0.36	2.553	− 14.04
308.15	2.545	2.548	− 0.13	2.248	11.67
313.15	3.009	3.012	− 0.09	3.015	− 0.21
*w*_3_ = 0.0200					
298.15	2.161	2.163	− 0.05	2.165	− 0.16
303.15	2.632	2.634	− 0.07	2.926	− 11.16
308.15	2.940	2.939	0.04	2.624	10.73
313.15	3.334	3.333	0.01	3.325	0.26
*w*_3_ = 0.0500					
298.15	2.597	2.593	0.16	2.592	0.18
303.15	2.944	2.944	0.00	3.205	− 8.88
308.15	3.210	3.213	− 0.09	2.942	8.34
313.15	3.521	3.523	− 0.05	3.520	0.03
*w*_3_ = 0.0700					
298.15	2.908	2.907	0.02	2.917	− 0.31
303.15	3.461	3.462	− 0.02	3.576	− 3.32
308.15	3.584	3.583	0.03	3.462	3.40
313.15	3.929	3.930	− 0.01	3.924	0.12
*w*_3_ = 0.1000					
298.15	3.094	3.093	0.06	3.051	1.40
303.15	3.834	3.817	0.44	4.206	− 9.70
308.15	4.208	4.208	0.00	3.845	8.63
313.15	4.349	4.355	− 0.13	4.348	0.02
*w*_3_ = 0.1500					
298.15	3.958	3.941	0.43	4.026	− 1.73
303.15	4.151	4.151	− 0.01	4.951	− 19.28
308.15	4.940	4.921	0.40	4.154	15.91
313.15	5.167	5.157	0.19	5.171	− 0.08
*w*_3_ = 0.2000					
298.15	4.309	4.319	− 0.23	4.336	− 0.62
303.15	4.778	4.787	− 0.19	5.355	− 12.08
308.15	5.362	5.372	− 0.20	4.745	11.49
313.15	5.751	5.758	− 0.12	5.755	− 0.07

An ACP solubility value of 2.65 × 10^–3^ (mole fraction) at 298.15 K in the co-solvent system methanol + water with 0.1 methanol weight fraction has been reported by Muoza et al. [48]. Compared to this value and at the same temperature and weight fraction, the solubility of ACP was higher in all co-solvent [MEA]La, [DMEA]La and [TEA]La systems being highest for [MEA]La PIL. ACP was also substantially less soluble at 298.15 K in the co-solvent system propylene glycol + water with an 0.1 weight fraction of propylene glycol (*x*_*1*_^*PG*^ = 2.37 × 10^–3^) than in the co-solvent system containing [MEA]La, [49]. ACP's solubility in solvent mixtures like aqueous ethanol and dioxan has been the subject of some reports [50–52]. Furthermore, the order of experimental solubility enhancement is [MEA]La > [DEA]La > [TEA]La. The solubility increase is due to the numerous factors, including solute–solvent interactions, polarity, hydrogen bonding interactions, enthalpy of fusion, and melting point. [26, 53]. The presence of hydrogen bonding interactions between ACP acting as the hydrogen bond acceptor, and the PILs as the hydrogen bond donor could explain the enhanced solubility of ACP in aqueous PIL co-solvent systems. In simpler terms, based on the experimental solubility data, the dissolution of ACP is directly influenced by the presence of ethanol amine groups and their ability to stablish strong hydrogen bonding interactions [26, 54]. These interactions could be mainly from the OH groups of ethanolamine (ETA) as NH3^+^ will be having electrostatic interaction with the carboxylic group of these acids. Notably, a liquid phase was only formed when ethanolamine was mixed with carboxylic acids in a 1:1 molar ratio, mirroring the composition of many protic ionic liquids (PILs) and deep eutectic solvents (DESs) generated through hydrogen-bond interactions between two equivalents of a hydrogen bond donor and one equivalent of a hydrogen bond acceptor. This raises the question of whether hydrogen-bond interactions between ethanolamine molecules, with the former acting as a hydrogen-bond acceptor (HBA) and the latter as a hydrogen-bond donor (HBD), could lead to the formation of PILs or DESs liquids at room temperature. Partial atom-to-atom radial distribution functions (pRDFs) indicated the presence of hydrogen bonding between the carboxyl group of the carboxylic acid (-COOH) and both the primary amine (-NH_2_) and hydroxyl group (-OH) of ETAH + .[55]. The enhanced solubility of ACPs in systems containing PILs is attributed to the presence of diverse intermolecular interactions, including strong ion–dipole interactions, dipole–dipole interactions, and hydrogen bonding [56].

### Modeling results

The experimental solubility data were further analyzed using various thermodynamic models, including the Modified Apelblat-Jouyban-Acree, Van’t Hoff-Jouyban-Acree, Wilson, and *e*-NRTL models, with the latter two serving as local composition models. The obtained results and corresponding model parameters are presented in Tables 4, 5, 6 and 7. Additionally, Table 8 summarizes the average relative deviation percent (ARD%) for each model's correlation performance. For the system containing [MEA]La, the adequacy of the models in predicting ACP solubility in aqueous PIL solutions within the temperature range of 298.15 to 313.15 K followed the order: Wilson > *e*-NRTL > Modified Apelblat-Jouyban-Acree > Van’t Hoff-Jouyban-Acree. Similarly, for the [DEA]La and [TEA]La co-solvent systems, the order of model adequacy was: Wilson > *e*-NRTL > Van’t Hoff-Jouyban-Acree > Modified Apelblat-Jouyban-Acree.

**Table 4:** The Modified Apelblat-Jouyban-Acree model's parameters for the ACP in the investigated systems

ACP (1) + [MEA]La (2) + (water) (3)									
*T* / K	*A*_1_	10^3^ *B*_1_	*C*_1_	*A*_2_	10^4^ *B*_2_	10^3^ *C*2	10^–3^ *J*_0_	10^–4^ *J*_1_	10^–4^ *J*_2_
298.15	0.001	− 0.313	− 0.286	− 5.258	− 2.077	− 0.146	0.258	0.241	0.405
303.15	0.026	24.000	− 0.756	− 3.367	− 1.527	− 0.428	− 1.332	− 892.030	0.070
308.15	− 0.288	− 0.078	− 2.019	− 5.374	− 1.298	− 0.047	0.016	− 1.105	− 0.836
313.15	12.202	− 6.498	222.700	− 5.502	− 0.121	0.001	− 0.007	− 51.950	− 16.950

ACP (1) + [DEA]La (2) + (water) (3)									
*T* / K	*A*_1_	10^5^ *B*_1_	*C*_1_	*A*_2_	10^5^ *B*_2_	10^4^ *C*2	10^–5^ *J*_0_	10^–5^ *J*_1_	10^–4^ *J*_2_
298.15	0.155	− 1.464	− 27.386	− 6.214	− 18.050	1.167	0.878	0.678	2.843
303.15	37.765	− 1.424	128.990	− 5.849	− 16.850	1.133	− 4.350	− 2.851	− 8.558
308.15	0.385	1.707	360.480	− 5.599	− 3.898	0.579	− 0.118	− 7.957	− 25.160
313.15	0.385	1.707	353.834	− 5.565	− 3.898	0.579	− 0.119	− 8.001	− 25.420

ACP (1) + [TEA]La (2) + (water) (3)									
*T* / K	*A*_1_	10^5^ *B*_1_	*C*_1_	*A*_2_	10^4^ *B*_2_	10^4^ *C*2	10^–5^ *J*_0_	10^–5^ *J*_1_	10^–5^ *J*_2_
298.15	0.626	3.529	82.087	− 6.214	− 12.220	0.145	− 2.626	− 1.798	− 0.576
303.15	− 193.290	− 1.465	1.289	− 6.122	− 11.950	1.158	1.048	0.756	0.281
308.15	− 824.550	− 2.930	5.144	− 5.927	− 30.650	2.317	4.475	2.883	0.856
313.15	− 0.001	− 1.470	52.840	− 5.676	− 29.617	1.157	− 1.879	− 1.478	− 0.578

**Table 5:** The Van't Hoff-Jouyban-Acree mode parameters for the ACP in the investigated systems

ACP (1) + [MEA]La (2) + (water) (3)							
*T* / K	*A*_1_	10^3^*B*_1_	*A*_2_	10^3^*B*_2_	*J*_0_	10^–4^ *J*_1_	10^–4^ *J*_2_
298.15	− 1.632	0.813	− 6.090	0.594	0.872	0.241	0.405
303.15	− 4.293	7.000	− 5.813	4.750	− 1.851	− 0.089	0.070
308.15	− 11.860	0.203	− 5.646	0.148	0.054	− 1.105	− 0.836
313.15	− 10.313	0.102	− 5.588	0.074	0.013	− 0.970	− 0.739

ACP (1) + [DEA]La (2) + (water) (3)							
*T* / K	10^−3^*A*_1_	*B*_1_	*A*_2_	*B*_2_	10^–4^ *J*_0_	10^–4^ *J*_1_	10^–4^ *J*_2_
298.15	− 0.200	0.054	− 6.216	0.038	11.240	8.437	3.369
303.15	0.772	0.027	− 5.848	0.019	− 43.360	− 28.420	− 8.529
308.15	1.830	0.027	− 5.614	0.019	− 104.900	− 70.500	− 22.280
313.15	− 0.222	0.054	− 5.724	0.038	12.670	8.434	2.762

ACP (1) + [TEA]La (2) + (water) (3)							
*T* / K	*A*_1_	*B*_1_	*A*_2_	*B*_2_	10^–6^ *J*_0_	10^–5^ *J*_1_	10^–4^ *J*_2_
298.15	471.155	0.006	− 6.213	0.005	− 0.264	− 1.809	− 5.797
303.15	2191.000	0.053	− 5.953	0.038	− 1.234	− 8.268	− 25.950
308.15	− 682.989	0.053	− 5.920	0.038	0.383	2.450	7.174
313.15	303.657	0.107	− 5.676	0.077	− 0.188	− 1.478	− 5.780

**Table 6:** The Wilson parameters of wilson model for the ACP in the investigated systems

*T* / K	10^5^* Λ*_*wd*_	*Λ*_*dw*_	10^3^*Λ*_*Cad*_	*Λ*_*dCa*_	10^4^*Λ*_*Caw*_	10^3^*Λ*_*wCa*_
ACP (1) + [MEA]La (2) + (water) (3)						
298.15	− 0.043	3.442	4.225	0.076	− 1.213	11.000
303.15	1.382	2.932	4.249	0.053	− 1.213	9.571
308.15	1.389	2.764	4.243	0.063	− 1.213	15.000
313.15	1.388	2.603	4.250	0.076	− 1.213	25.000

*T* / K	10^5^* Λ*_*wd*_	*Λ*_*dw*_	10^3^*Λ*_*Cad*_	*Λ*_*dCa*_	10^4^*Λ*_*Caw*_	10^3^*Λ*_*wCa*_
ACP (1) + [DEA]La (2) + (water) (3)						
298.15	96.280	3.024	4.070	0.101	− 1.211	2.108
303.15	1.976	2.931	5.229	0.038	− 1.219	7.560
308.15	2.078	2.763	4.685	0.015	− 320.000	2.280
313.15	− 1.942	2.602	4.152	0.043	− 1.212	13.000

*L*	10^5^* Λ*_*wd*_	*Λ*_*dw*_	*Λ*_*Cad*_	10^5^*Λ*_*dCa*_	10^4^*Λ*_*Caw*_	10^3^*Λ*_*wCa*_
ACP (1) + [TEA]La (2) + (water) (3)						
298.15	− 1.589	3.105	− 0.065	− 1.705	− 1.212	− 3.897
303.15	− 2.294	2.934	− 0.067	− 1.594	− 1.213	− 3.883
308.15	− 16.530	2.762	− 0.071	− 9.193	− 1.228	− 5.820
313.15	− 16.530	2.601	− 0.071	− 9.186	− 1.228	− 6.000

w = water, d = drug (ACP), Ca = cation (2-hydroxyethylammonium (MEA) Bis-2-hydroxyethylammonium (DEA) Tris-2-hydroxyethylammonium (TEA) and anion (Lauric acid)

**Table 7:** The *e*-NRTL mode parameters for the ACP in the investigated systems.

*T* / K	10^4^ *Δg*_*wd*_	*Δg*_*dw*_	10^3^*Δg*_*Cad*_	10^3^*Δg*_*dCa*_	10^3^*Δg*_*Caw*_	10^3^*Δg*_*wCa*_
ACP (1) + [MEA]La (2) + (water) (3)						
298.15	0.924	2.001	4.144	1.711	− 0.048	− 0.400
303.15	1.083	1.022	6.794	0.572	− 3.693	− 0.607
308.15	1.104	1.385	4.354	4.778	− 4.451	− 5.550
313.15	1.140	1.019	5.096	0.585	− 4.545	− 0.621

*T* / K	10^4^*Δg*_*wd*_	*Δg*_*dw*_	10^3^*Δg*_*Cad*_	10^3^*Δg*_*dCa*_	10^3^*Δg*_*Caw*_	*Δg*_*wCa*_
ACP (1) + [DEA]La (2) + (water) (3)						
298.15	0.928	2.151	4.541	1.946	− 0.045	− 412.982
303.15	0.845	0.360	2.195	− 0.349	1.589	364.955
308.15	1.116	2.464	4.830	0.366	− 4.215	− 403.353
313.15	1.112	2.460	2.487	0.372	− 6.121	− 409.642

*L*	10^4^* Δg*_*wd*_	10^3^*Δg*_*dw*_	10^3^*Δg*_*Cad*_	10^3^*Δg*_*dCa*_	10^3^*Δg*_*Caw*_	10^3^*Δg*_*wCa*_
ACP (1) + [TEA]La (2) + (water) (3)						
298.15	1.850	19.424	4.272	1.380	− 13.690	− 1.822
303.15	1.094	35.310	3.368	1.157	− 4.974	− 1.465
308.15	1.076	35.302	3.377	1.155	− 4.994	− 1.463
313.15	1.107	35.317	3.361	1.150	− 4.961	− 1.462

w = water, d = drug (ACP), Ca = cation cation (2-hydroxyethylammonium (MEA) Bis-2-hydroxyethylammonium (DEA) Tris-2-hydroxyethylammonium (TEA)) and anion (Lauric acid)

**Table 8:** The calculated activity coefficient of ACP, ln $\gamma_{1}^{{}}$, based on Wilson model in aqueous PILs solutions at different temperatures respectively

PILs weight fraction	*T* = 298.15 K	*T* = 303.15 K	*T* = 308.15 K	*T* = 313.15 K
ACP (1) + [MEA]La (2) + (water) (3)				
0.0000	2.8782	3.1888	3.2289	3.2234
0.0200	2.4783	2.7698	2.8317	2.9398
0.0500	2.3057	2.6438	2.7629	2.8614
0.0700	2.1317	2.4737	2.6216	2.7119
0.1000	2.0105	2.4243	2.477	2.6029
0.1500	1.8845	2.2632	2.351	2.4295
0.2000	1.7674	2.1845	2.2624	2.3331
ACP (1) + [DEA]La (2) + (water) (3)				
0.0000	3.2139	3.1898	3.2299	3.2243
0.0200	2.9108	2.8485	2.8973	3.0132
0.0500	2.6667	2.7223	2.7983	2.8848
0.0700	2.5829	2.6167	2.7360	2.8186
0.1000	2.4747	2.5562	2.5980	2.6664
0.1500	2.3665	2.4671	2.5461	2.5974
0.2000	2.2833	2.3452	2.4221	2.5041
ACP (1) + [TEA]La (2) + (water) (3)				
0.0000	3.2111	3.1868	3.2286	3.2237
0.0200	3.0520	3.0278	3.0861	3.1223
0.0500	2.8705	2.9168	2.9970	3.0669
0.0700	2.7560	2.7547	2.8879	2.9577
0.1000	2.6943	2.6569	2.7271	2.8550
0.1500	2.4519	2.5731	2.5706	2.6860
0.2000	2.3602	2.4305	2.4828	2.5757

**Fig. 6:**
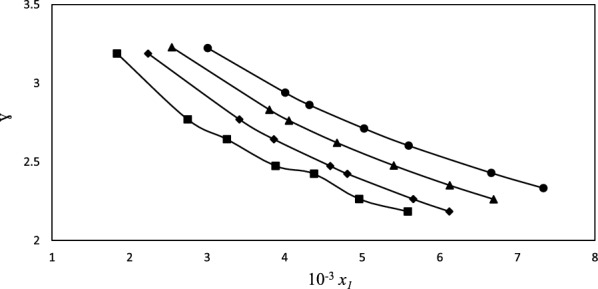
The following is the relationship between the solubility of ACP and the calculated activity coefficient in the aqueous solution containing [MEA]La from the Wilson model at various temperatures; 298.15(■), 303.15 (♦), 308.15 (▲), 313.15 (●)

Activity coefficients play a pivotal role in understanding and quantifying nonideal effects in solubility behavior. They bridge the gap between ideal and real-world solutions, allowing us to explore deviations and make accurate predictions. in this regard, importance of considering activity coefficients to describe the nonideal effects in the solubility behavior of the studied mixtures. Table 9 and Fig. 6 presents the calculated activity coefficients ($\gamma_{1}^{{}}$) for ACP in the systems using the Wilson model for the [MEA]La co-solvent system at different temperatures. The solubility of ACP is directly related to its activity coefficient. When the activity coefficient is high, the ACP molecules are less likely to escape into the solvent, resulting in lower solubility. Conversely, when the activity coefficient is low, the ACP molecules are more prone to disperse into the solvent, leading to higher solubility. according to the mentioned explanation and activity coefficent values, it could observed that $\gamma_{1}^{{}}$ values decrease with an increase in the weight fraction of the protic ionic liquids present in the systems. The results show that as the experimental solubility increases with temperature, the activity coefficient decreases, indicating enhanced interactions between ACP and co-solvent. This observation confirms that the activity coefficient decreases as a result of increased interactions [57].

**Table 9:** The average relative deviation percent (ARD%) for the ACP's solubility in the aqueous PILs solutions at a *T */ K^a^ = 298.15 to 313.15 and a pressure of P^a^ = 866 hPa, as determined by the utilized models

	*ARD%*			
*T*/K	Modified Apelblat-Jouyban-Acree	Van't Hoff-Jouyban-Acree	Wilson	*e*-NRTL
	ACP (1) + [MEA]La (2) + (water) (3)			
298.15	1.484	1.495	0.099	0.564
303.15	1.819	1.827	0.117	0.689
308.15	2.019	2.148	0.097	0.594
313.15	1.358	1.883	0.082	0.441
Average	1.690	1.838	0.099	0.572
	ACP (1) + [DEA]La (2) + (water) (3)			
298.15	0.502	0.550	0.073	0.448
303.15	1.027	0.650	0.098	0.415
308.15	0.993	0.888	0.078	0.345
313.15	2.562	1.647	0.058	0.287
Average	1.271	0.934	0.077	0.456
	ACP (1) + [TEA]La (2) + (water) (3)			
298.15	1.933	1.681	0.177	0.793
303.15	2.619	1.198	0.156	0.264
308.15	1.026	1.111	0.127	0.218
313.15	0.696	1.409	0.089	0.113
Average	1.568	1.350	0.137	0.331

^*a*^ Standard uncertainty u(*T*) = 0.01 K

^b^ Standard uncertainty u*(P)* = 10 hPa

### Thermodynamic properties of dissolution results

**Fig. 7:**
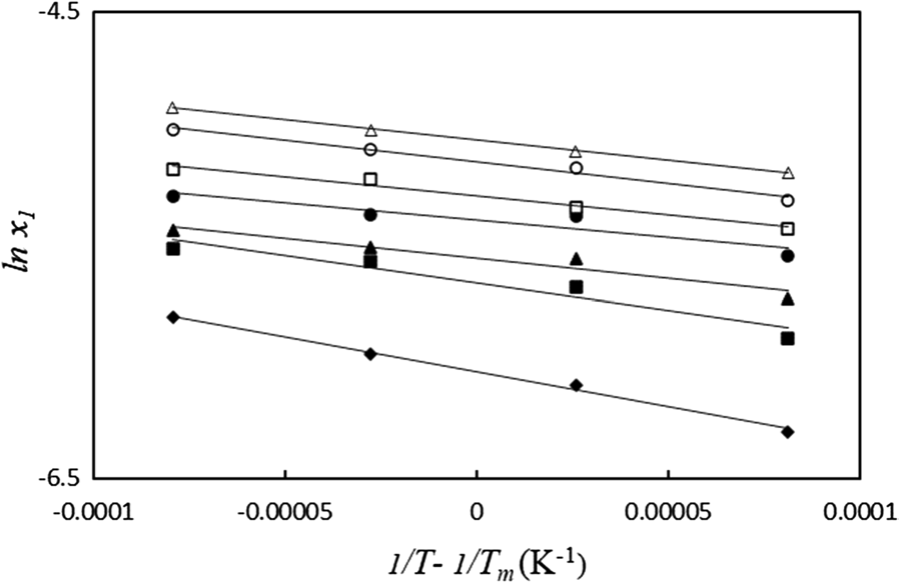
Plot of *lnx*_*1*_ vs (*1/T- 1/T*_*hm*_); in aqueous [MEA]La solutions at different weight fraction of the PILs (*w*_*PILs*_*)*: 0.0000(♦), 0.0200 (■), 0.0500 (▲), 0.0700 (●), 0.1000 (□), 0.1500 (o), 0.2000 ($\vartriangle$)

**Fig. 8:**
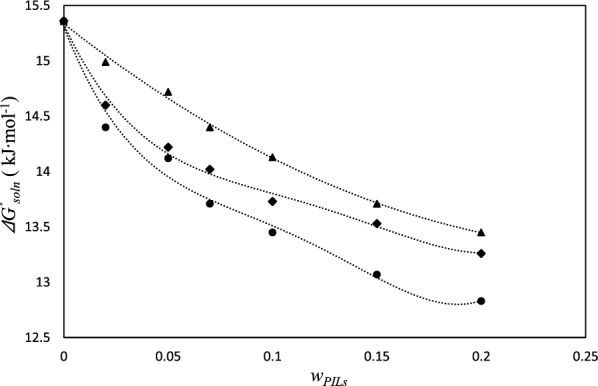
The $\Delta G_{so\ln }^{^\circ }$ values relative to dissolution process of ACP in the aqueous PILs solutions at 305.548 K, [MEA]La (●), [DEA]La (♦), [TEA]La (▲)

Figure 7 presents the van’t Hoff plots of ACP solubility data for the [MEA]La system. Fom the results, the value of the thermodynamic parameters of dissolution ($\Delta H_{so\ln }^{o}$, $T_{m} \Delta S_{so\ln }^{o}$ and $\Delta G_{so\ln }^{o}$) were calculated and listed in Table 10. The positive values of (ΔG^0^_soln_) and ($\Delta H_{so\ln }^{o}$) show an endothermic process for the dissolution of ACP in these aqueous PIL systems. The ΔG^0^_soln_ values were smaller as the weight fraction of PILs increased as can be seen in Fig. 8. Moreover, the $T_{hm} \Delta S_{so\ln }^{o}$ values are positive for the dissolution process. The relative contributions of enthalpy and entropy to the standard molar Gibbs free energy of dissolution of ACP in PIL based systems was expressed in $\xi_{H}$ and $\xi_{TS}$ (eqs. 17 and 18). The relatively lower entropy values compared to the enthalpy of dissolution suggest that the enthalpy of dissolution plays a more significant role in the ACP dissolution process in these aqueous PIL systems [58].

**Table 10:** Thermodynamic functions for dissolution process at different weight fractions of PIL (w_3_) ^a^ at mean temperature ^b^

*w*_*3*_	$\Delta H_{{so\,\,{\text{ln}}\,}}^{ \circ }$/kJ∙mol^−1^	$T_{M} \Delta H_{{so\,\,{\text{ln}}\,}}^{ \circ }$/kJ∙mol^−1^	$\Delta G_{{so\,\,{\text{ln}}\,}}^{ \circ }$/kJ∙mol^−1^	$\xi_{H}$	$\xi_{TS}$
ACP (1) + [MEA]La (2) + (water) (3)					
0.0000	24.933	9.576	15.356	72.250	27.750
0.0200	19.284	4.888	14.395	79.777	20.223
0.0500	13.991	− 0.131	14.122	99.076	0.924
0.0700	12.336	− 1.378	13.714	89.953	10.047
0.1000	13.299	− 0.148	13.447	98.898	1.102
0.1500	15.005	1.931	13.074	88.600	11.400
0.2000	14.056	1.225	12.831	91.982	8.018
ACP (1) + [DEA]La (2) + (water) (3)					
0.0000	24.933	9.576	15.356	72.250	27.750
0.0200	20.612	6.015	14.598	77.411	22.589
0.0500	14.671	0.450	14.221	97.024	2.976
0.0700	13.237	− 0.784	14.021	94.407	5.593
0.1000	16.569	2.841	13.727	85.361	14.639
0.1500	14.041	0.514	13.528	96.472	3.528
0.2000	14.515	1.260	13.256	92.015	7.985
ACP (1) + [TEA]La (2) + (water) (3)					
0.0000	24.933	9.576	15.356	72.250	27.750
0.0200	21.931	6.937	14.994	75.970	24.030
0.0500	15.535	0.819	14.716	94.992	5.008
0.0700	14.600	0.198	14.402	98.659	1.341
0.1000	17.384	3.253	14.131	84.235	15.765
0.1500	15.113	1.400	13.713	91.522	8.478
0.2000	15.242	1.793	13.449	89.476	10.524

^*a*^ Standard uncertainty u is u(*w*_*3*_) = 0.0002

^b^Standard uncertainty u(*T*) = 0.01 K

## Conclusions

In the present study, the effect of  three protic ionic liquids [MEA]La, [DEA]La, and [TEA]La  on the experimental aqueous solubility of acetaminophen (ACP) drug at different temperatures and weight fractions of the PILs was measured. The results presented that enhancement in ACP solubility by increasing the temperature and weight fraction of the PILs . Due to its strong hydrogen bonding interactions, [MEA]La revealed the greatest solubility improvement. Besides, various thermodynamic models, including the Wilson, *e*-NRTL and the emprical models, Van’t Hoff-Jouyban-Acree, and Modified Apelblat-Jouyban-Acree were applied to correlate the experimental aquous ACP solubility data. The performance for the system containing [MEA]La follow the trend for activity coefficient and empirical models, respectively: the Wilson > *e*-NRTL and Modified Apelblat—Jouyban—Acree > Van’t Hoff—Jouyban—Acree. On the other hand, [DEA]La and [TEA]La PILs followed slightly different trend for activity coefficient models and empirical respectively: the Wilson >* e*-NRTL and Van’t Hoff–Jouyban–Acree > Modified Apelblat–Jouyba–Acree. Subsequently, an overview of the thermodynamic dissolution process of ACP in the systems under investigation was carried out. The outcomes demonstrated that enthalpy drives the endothermic dissolving process in all utilized co-solvents.

### Supplementary Information

Supplementary file 1.

## Data Availability

The datasets utilized and/or analyzed during this study are available from the corresponding author on reasonable request.

## Abbreviations

(VOCs): Volatile organic compounds
(LMMs): Low-melting mixtures
(PILs): Protic ionic liquids
(ACP): Acetaminophen
[MEA]La: 2-Hydroxyethylammonium laurate
[DEA]La: Bis(2-hydroxyethyl)ammonium laurate
[TEA]La: Tris(2-hydroxyethyl)ammonium laurate
