# Supplementary material for: Effect of protic surfactant ionic liquids based on ethanolamines on solubility of acetaminophen at several temperatures: measurement and thermodynamic correlation
Source: BMC Chem. 2024 Jul 25;18(1):136. doi: 10.1186/s13065-024-01243-x (PMC11270923; doi:10.1186/s13065-024-01243-x)
Supplement: Supplementary file 1 [file 13065_2024_1243_MOESM1_ESM.docx]

**Supporting Information**

**Effect of protic surfactant ionic liquids based on ethanolamines on solubility of acetaminophen at several temperatures: measurement and thermodynamic correlation**

**Parisa Akbarzadeh Gondoghdi^1^, Hemayat Shekaari^1^^[[1]](#footnote-1)^*, Masumeh Mokhtarpour^1,2^, Mirhesam Miraghazadeh Sardroud^1^, Ramin Afkari^1^, Mohammad Khorsandi^1^**

^1^ Department of Physical Chemistry, University of Tabriz, Tabriz, Iran.

^2^ Research Center for Bioscience and Biotechnology, University of Tabriz, Tabriz, Iran.

**Availability of data and materials**

The datasets utilized and/or analyzed during this study are available from the corresponding author on reasonable request.


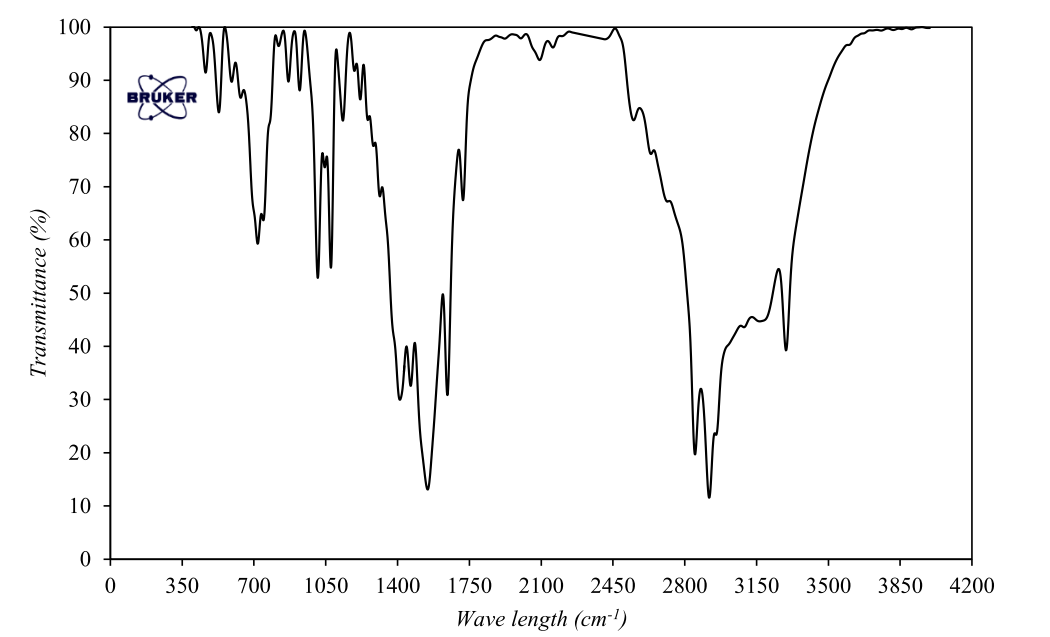


**Figure S1.** FT-IR spectrum of 2-hydroxyethylammonium laurate [MEA]La


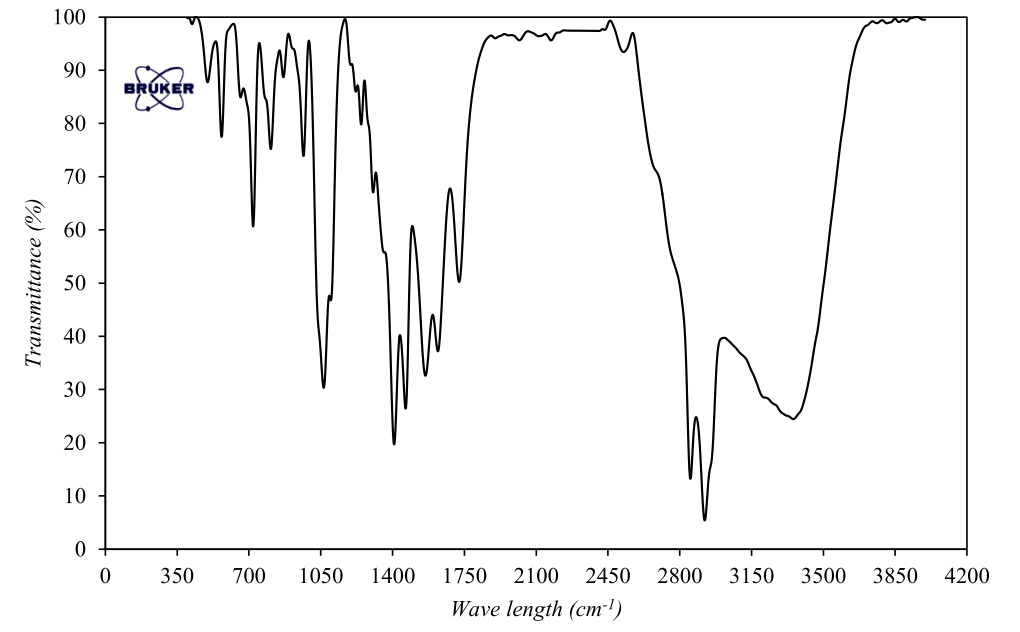


**Figure S2.** FT-IR spectrum of Bis-2-hydroxyethylammonium Laurate [DEA]La


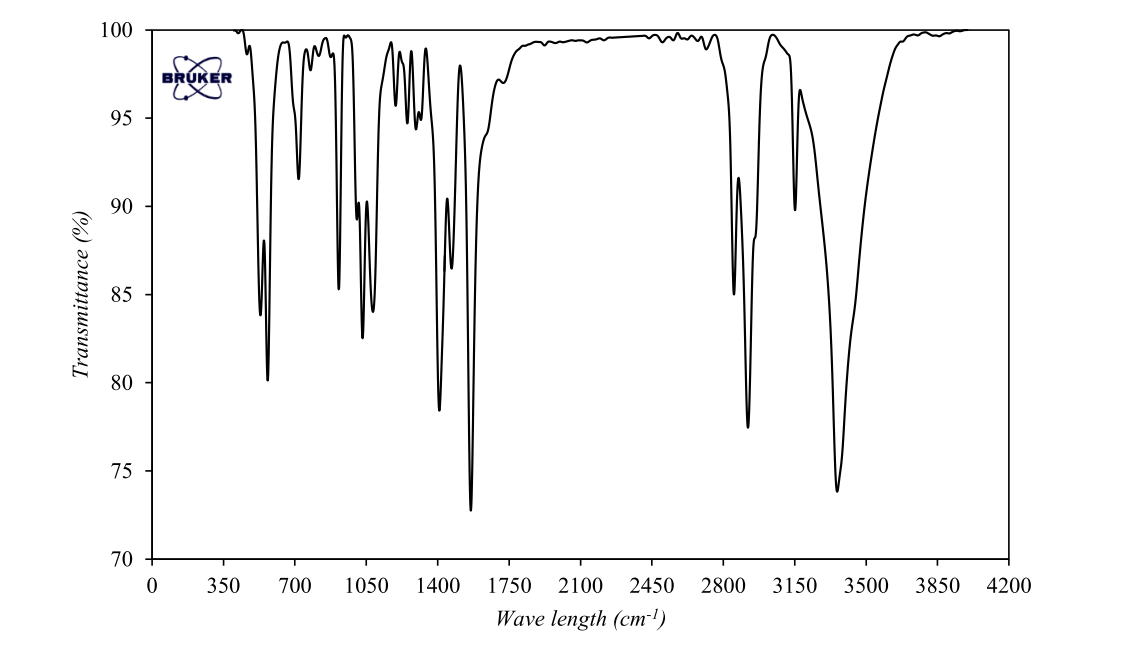


**Figure S3.** FT-IR spectrum of Tris-2-hydroxyethylammonium Laurate [TEA]La.


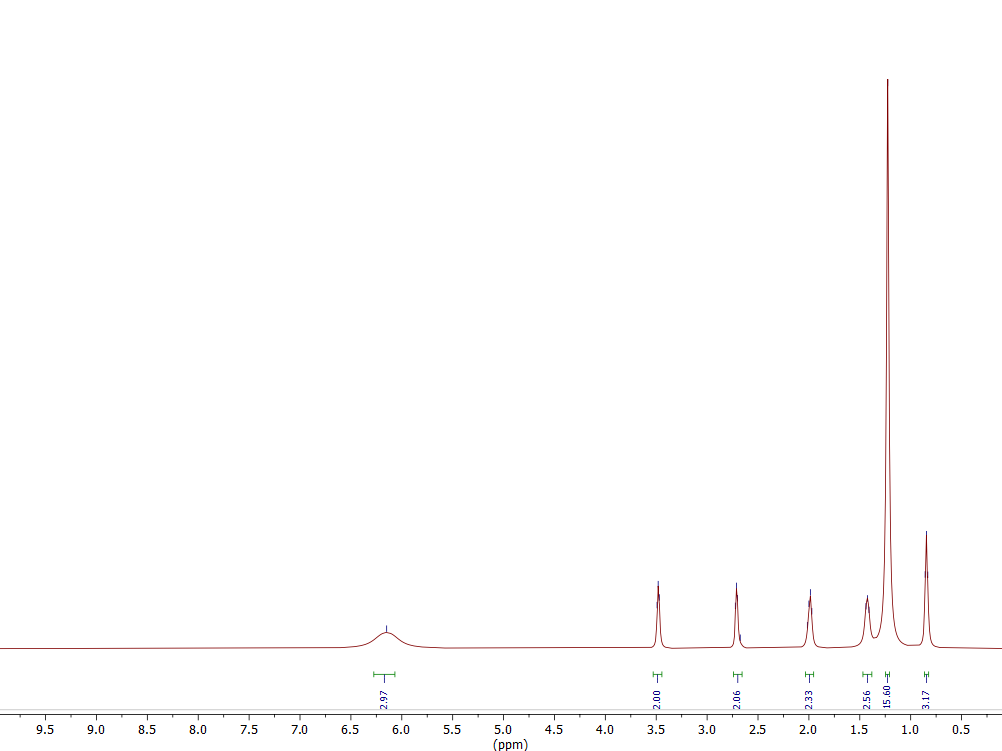


**Figure S4.** 1H NMR of 2-hydroxyethylammonium Laurate [MEA]La.


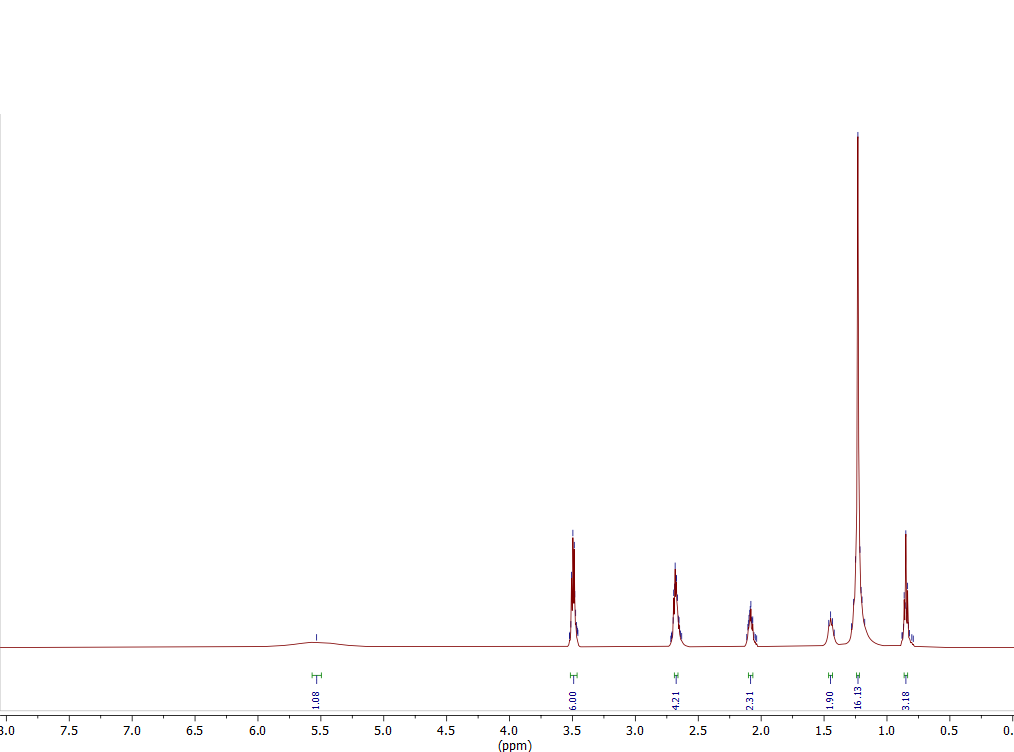


**Figure S5.** 1H NMR of Bis-2-hydroxyethylammonium Laurate [DEA]La.


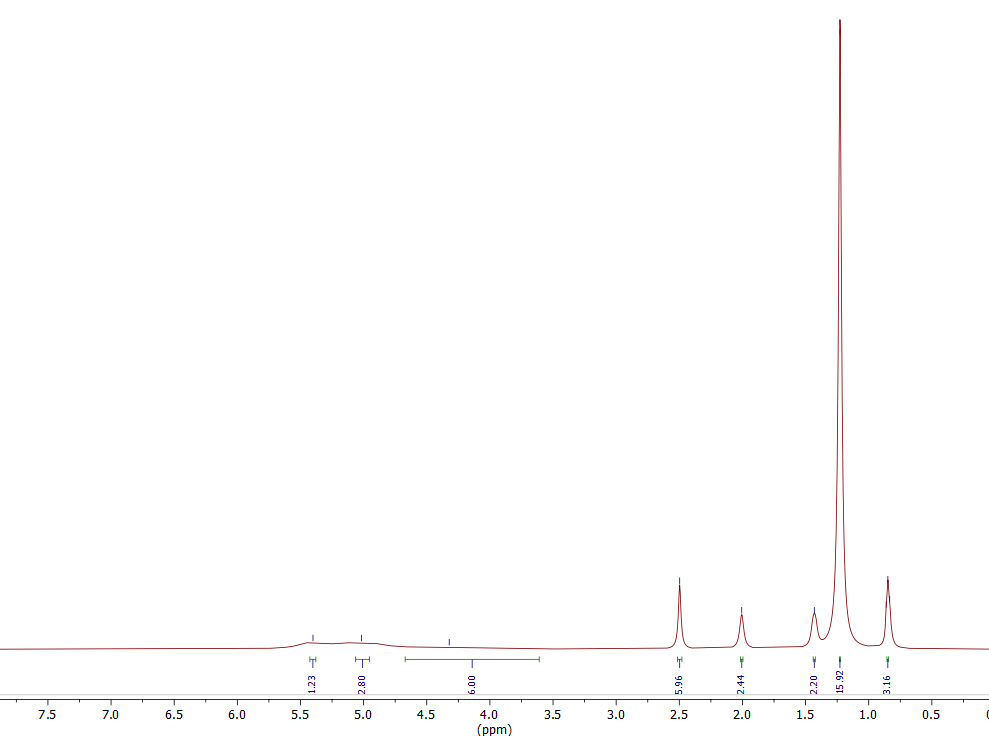


**Figure S6.** 1H NMR of Tris-2-hydroxyethylammonium Laurate [TEA]La.

1. * *Corresponding author. Tel.: +*98-41-33393094. Fax: +98-41-33340191.

   E-mail address: hemayatt@yahoo.com (H. Shekaari). [↑](#footnote-ref-1)
